# Energy drinks may affect the ovarian reserve and serum anti-mullerian hormone levels in a rat model

**DOI:** 10.4274/tjod.galenos.2020.07347

**Published:** 2021-03-12

**Authors:** Erkan Elçi, Gülhan Güneş Elçi, Numan Çim, İbrahim Aras, Sena Sayan, Recep Yıldızhan

**Affiliations:** 1 University of Health Sciences Turkey, Ümraniye Training and Research Hospital, Clinic of Obstetrics and Gynecology, İstanbul, Turkey; 2 University of Health Sciences Turkey, Sancaktepe Training and Research Hospital, Clinic of Obstetrics and Gynecology, İstanbul, Turkey; 3 İstanbul Bilim University Faculty of Medicine, Department of Obstetrics and Gynecology, İstanbul, Turkey; 4 Yüzüncü Yıl University Faculty of Medicine, Department of Pathology, Van, Turkey; 5 Marmara University Faculty of Medicine, Department of Obstetrics and Gynecology, İstanbul, Turkey

**Keywords:** Energy drinks, anti-mullerian hormone, ovarian reserve, antral follicles

## Abstract

**Objective:**

Energy drinks have an impact on concentration levels, physical performance, speed of reaction, and focus, but these drinks cause many adverse effects and intoxication symptoms. The main goal of this study was to determine the effect of energy drink consumption on ovarian reserve and serum anti-mullerian hormone (AMH) levels.

**Materials and Methods:**

Female Wistar albino rats (n=16) were included and randomized into two groups (n=8). Serum AMH levels were checked before and after energy drinks were given. Eight weeks later, the ovaries and uteruses of the rats were analyzed histopathologically. The number of follicles in the ovaries was counted.

**Results:**

The total number of the preantral plus small antral follicles, which show the ovarian reserve, was decreased at the end of eight weeks in both the control group and the energy drink group. There was a statistical difference between them (p=0.021). Also, there was a statistically significant difference in the initial/final AMH (ng/mL) reduction levels between the control group and the energy drink group (p=0.002). AMH levels were decreased more in the energy drink group.

**Conclusion:**

The consumption of energy drinks can lead to a decrease in ovarian reserve and AMH values and may cause weight gain.

**PRECIS:** Energy drinks became much more popular. These drinks are categorized as sugar sweetened beverages. People need to be careful about the energy drink consumption in terms of reproductive health.

## Introduction

Energy drinks (EDs) have become much more popular since the 1960s^(1)^. These drinks are categorized as sugar-sweetened beverages. They also contain caffeine, taurine, glucuronolactone, and other vitamins and mineral additives^(2)^. EDs have been the fastest growing area of the beverage industry to date. In advertisements, companies assert that these drinks have a good impact on concentration levels, physical performance, speed of reaction, focus, and wellness^(3)^. Despite the positive effects, these drinks cause many cardiovascular adverse effects and intoxication symptoms causing concerns about the health of the consumers^(4)^. From 2001 to 2008, the level of ED consumption in adolescents and adults was estimated to have increased from 24% to 56%, causing greater concern^(5)^.

EDs have been completely prohibited or sold in low caffeine forms in some countries because of their adverse effects. Turkey is one of the countries that prohibit the high caffeine forms^(6)^, but in most countries, EDs are qualified as nutrient support and there are no restrictions^(6)^. The United States of America Food and Drug Administration updated the classification of EDs as dietary supplements^(6)^. The percentages of each ingredient are different in every brand^(6)^. The most prevalent ingredients of EDs are caffeine, taurine, glucuronolactone, vitamin B complex, and other herbal stimulants, most of which have been studied little^(6)^.

In terms of the reproductive system, studies have shown that caffeine probably decreases the estrogen and progesterone levels in the luteal phase and increases the risk of a shorter menstrual cycle (<25 days)^(7)^. However, studies have also shown that EDs have little effect on ovarian aging with their ovulation stimulant effect^(7)^.

The second major component in EDs is taurine^(8)^, which is the richest amino acid in mammalian cells and it plays a major role in many important biologic events. It acts as a neurotransmitter, and an osmoregulatory and antioxidant agent in many tissues^(8)^. In female rats, taurine exists in uterine tissue, uterine fluid, ovarian theca cells, and in cells that are responsible for androgen synthesis^(8)^. Taurine is a popular agent that is presented as a performance-enhancing agent and accepted to be safe, but many researchers think that the effects of high-dose taurine in EDs should be studied^(9)^.

Other components are carbohydrates (glucuronolactone) and vitamin B complex. Although they show the effects of vitamins to caffeine and taurine, they act as coenzymes^(10)^. Carbohydrates, on the other hand, exist to provide energy to increase metabolism. However, consuming carbohydrate beverages may increase the risk of metabolic syndrome and weight gain may cause infertility^(9)^. It should be remembered that obese and overweight individuals have reduced fertility^(11)^.

In this study, we aimed to analyze the effect of EDs on ovarian reserve in rats by examining AMH levels and ovarian histopathology.

## Materials and Methods

The study was approved by the local ethics committee of the Yüzüncü Yıl University Faculty of Medicine Department in Van, Turkey, for the use of laboratory animals and was performed at the Experimental Surgery Training and Education Center at the same hospital (approval number: 2015-HIZ-TF290).

### Animal Maintenance and Treatment

In this study, sixteen healthy adult female albino rats (8 to 10 weeks old) weighing 190±10 g were used. The animals were kept according to the institutional review board’s guidelines for animal care, in a 14-hour light cycle at controlled temperatures (22-28 °C), and food and water were available ad libitum. The water consumption of the rats was not recorded. The weight of the rats was recorded daily and the food they consumed was recorded weekly. After the acclimation period, the stages of the estrus cycles of the rats were evaluated by performing daily vaginal smears.

The rats were randomly assigned to two study groups (8 rats each). In the control group (group I), the rats were kept on a normal diet and given water for 8 weeks. In the ED group (group II), the rats were kept on a normal diet and given water plus a daily single dose of ED. The dosing was calculated in comparison with the surface area of humans and rats (3.9 mL/kg b.w.)^(12)^. A 250-mL can of commercially available ED (A-5330 Fuschl am See, Austria) was opened daily between 09:00 and 10:00 and approximately 0.7 mL was given orally for each rat via flexible oral gavage tubes. A single dose of the ED is roughly equivalent to the minimal human dose [1 can (250 mL)/day], but of course it varies according to the animal’s surface area. Each 100 mL of ED contains a mixture of water, taurine (0.4%) (400 mg), caffeine (0.032%) (320 mg/L), gluconolactone (0.24%), inositol, sucrose, glucose, sodium citrate, carbon dioxide niacin (8 mg), pantothenic acid (2 mg), vitamin B6 (2 mg), B12 (0.002 mg), caramel, riboflavin, and natural and artificial flavoring and coloring agents (these are listed ingredients on the label).

### Blood Sampling, Tissue Collection, and Histopathologic Analysis

After the acclimation at the beginning of the study (initial) and following the 8-week period (final), blood samples (1 mL) were obtained from the right jugular vein of each rat to measure the serum AMH levels under general anesthesia. The animals were anesthetized by administering 50 mg/kg 10% ketamine hydrochloride (Ketasol; Richter Pharma) and 5 mg/kg 2% xylazine (Rompun; Bayer Healthcare) intramuscularly. All blood samples were immediately centrifuged at 4000 *g* for 10 minutes, and the collected sera were transferred to Eppendorf tubes. The samples were then transferred on ice and kept at -80 °C in a deep freeze until analysis using an automatic enzyme-linked immunosorbent assay (ELISA)^(13)^ system with a commercially available kit (Cusabio Biotech Co, Wuhan, China). The AMH assay measured concentrations with an assay range of 0.2-15 ng/mL; the manufacturer-specific mean inter and intra-assay coefficient of variation (CV) was less than 15% (CV<15%). All samples and standards were assayed in duplicate as recommended in the catalogue of AMH.

The rats were sacrificed in estrus cycles using cervical dislocation and bilateral oophorectomies and hysterectomies were performed in all rats. Histologic ovarian and uterus tissue samples were evaluated by a single histopathologist who was blinded to the origin of the samples. The volumes of the ovaries and uteruses were measured under a microscope. Tissues were fixed in 10% formaldehyde for 72 hours, underwent routine tissue processing, and then embedded in paraffin wax. Four-micron-thick sections were taken from the tissues and the tissues were completely consumed. All sections were stained with hematoxylin and eosin. All sections were investigated under a light microscope (Zeiss Axioskop 40 Carl Zeiss Göttingen, Germany) and the pieces were photographed (AxioVision 3.1 Zeiss Axioplan 2 imaging Germany, Göttingen). These sections were evaluated for follicle counting, with one in four sections in the order of sections. The histologic examination method was performed according to the model of Durlinger et al.^(14,15)^.

**Figure 1:**
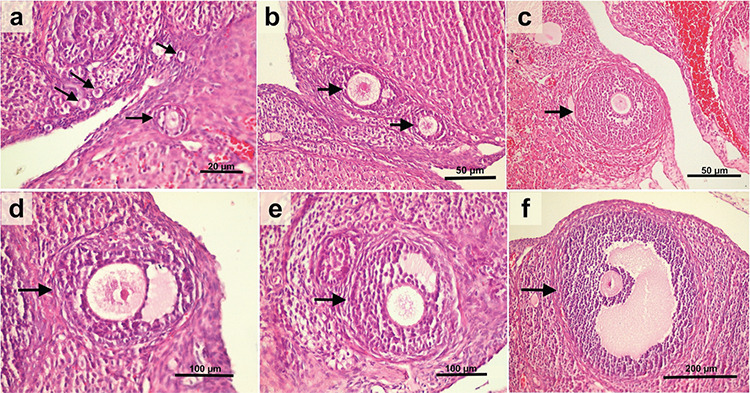
(a). Light micrograph showing primordial follicles (≤20 μm) (Bar=20), (b) and (c) arrowheads indicate preantral follicle (20-220 μm) (Bars=50), (d) and (e) arrowheads indicate antral follicle (221-310 μm) (Bars=100 μm), (f) arrowhead indicate large antral follicle (311-370 μm) (Bar=200 μm). hematoxylin and eosin

Primordial follicles are nongrowing follicles and consist of an oocyte partially or completely encapsulated by flattened squamous pregranulosa cells. Early primary follicles have initiated development and contain at least one cuboidal (enlarged) granulosa cell^(16)^. In addition, primordial follicles and early primary follicles, which can be distinguished by their size: follicles with a mean diameter less than or equal to 20 µm are classified as primordial follicles, and follicles with a mean diameter greater than 20 µm are classified as growing follicles [preantral (PA), small antral, and large antral follicles]^(14,15)^. The follicles were divided into four groups according to the average diameter determined by measuring two vertical diameters in the section where the oocyte nucleolus was^(14,15)^; primordial follicles (≤20 µm) (Figure 1a, PF (20-220 **µ**m) (Figure 1b,c), SF (221-310 **µ**m) (Figure 1d,e), and LF (311-370 **µ**m) (Figure 1f). Atretic follicles were excluded from the count.

Stereologic methods were used as the assessment method of ovarian and uterine volume measurements. Stereology is a method by which random and quantitative information is obtained by using random systematic sampling. From the stereologic analysis, the modified method of the dissector Cavalieri principle was used^(17)^. The endometrium and ovarian volume ratio was measured using the dotted area ruler given in the Shetereom Ver. 1.5 package program^(17,18)^.

Vaginal smears were taken at the same time daily in over 8 weeks. The length and layout of the estrous cycles were evaluated using vaginal smears. Dried smears were examined microscopically and the estrus cycle stage was determined according to the criteria of Allen^(19)^. No differences were found between the two groups of rats in either the length or the regularity of the estrous cycle; regular estrous cycles with a length of 4-5 days were found (results not shown).

The rats were weighed individually at the beginning and eight weeks later. The rats that drank the ED weighed more compared with those that drank water.

### Statistical Analysis

Descriptive statistics for the studied variables (characteristics) are presented as median, mean, standard deviation, minimum, and maximum values. The Mann-Whitney U test was performed to compare the groups. A statistical significance level was considered as 5% and the Statistical Package for the Social Sciences (SPSS) (Ver. 22) statistical program was used for all statistical computations.

## Result

There was statistical significance between the means of the weight changes of the two groups (p=0.002) (Table 1). The weekly consumed pellets of the rats showed a statistical difference between group I and group II in regards to food consumption at the end of the 8 weeks (p=0.001) (Table 1).

The uterus (n=16) and ovarian tissues (n=32) excised from the rats after fourteen cycles were evaluated morphologically. When the ovarian tissues of the rats that were given EDs were compared with the control group, their mean ovarian volume was smaller (10.78±2.9 mm^3^) but there was no statistically significant difference (p=0.99). The endometrium, myometrium, and serosa layers of the uteruses of both groups were histologically normal. Endometrial volumes (mm^3^) of both groups were evaluated in stereology in terms of endometrium thickness. The mean volume of endometrium in the ED group (38.83±21.2) was more than in the control group (29.28±14.48) (Table 2), but there was no statistically significant difference (p=0.565) (Table 2). According to these findings, the ED that was used in the experimental group did not affect the endometrium or other layers of the uterus.

The total follicles in the ovaries were evaluated. There was no statistically significant difference between the follicles of the two groups (p=0.283). In terms of ovarian reserve analysis, the number of PF, SF, and PA plus small antral follicles (PSF) were counted. Even though the means of PF and SF were decreased more in the subject group, there was no statistically significant difference between the two groups (p=0.026, p=0.057). Furthermore, the means of the total number of PSFs were decreased more in the subject group (160±30.9) than in the control group (133±28.6) and a statistical significance was shown between the two groups (p=0.021) (Table 3).

No statistically significant difference was seen between the mean levels of AMH taken from the jugular veins of the rats at the beginning and eight weeks later, but a statistically significant difference was shown between the means of decrease in the levels of AMH (p=0.002). The decrease was shown to be more in the subject group (Table 4).

## Discussion

Ovarian function is very important for reproductive health. Follicles play a key role in the reproductive function of the ovaries^(20)^. In the development of follicles, along with several local factors, systemic (hypothalamus and/or pituitary) mediators affect their functions at a certain stage^(20)^. AMH is the most important mediator indicating the functions of follicles and providing information about their reserves. PF and AF counts are responsible for the synthesis of this mediator^(20,21)^. It was reported that AMH levels were a better indicator for ovarian reserve than age, follicle-stimulating hormone (FSH), luteinizing hormone (LH), inhibin-B, and, estradiol (E_2_)^(21,22)^. AMH is a dimeric glycoprotein that belongs to the transforming growth factor family^(23)^. AMH protein expression starts immediately after the follicle recruitment and continues to the antral stage of the follicle^(22,24)^. PFs are the main source of folliculogenesis in the ovaries^(20)^. As soon as PF development begins, AMH plays a protective role by slowing down the rate of consumption of the local primordial follicle pool from the granulosa cells^(14)^. AMH also regulates the growth rate of follicles by inhibiting FSH-related follicle growth in the early antral period^(14)^. Three-quarters of all AMH is found in PFs and SA follicles^(22)^. In follicles without AMH in rats, preliminary estrus cycle loss was observed due to the rapid depletion of the primordial pool^(15)^. Loss of PFs causes irreversible infertility^(10,15)^.

The common active ingredients used in EDs are caffeine, taurine, sugar, and a vitamin complex. The biggest difference between these drinks and sugary drinks is that they contain caffeine and taurine.

Caffeine acts as a phosphodiesterase (PDE) inhibitor^(10)^. PDE 2 is the enzyme that destroys the signal path stimulant cyclic adenosine monophosphate (cAMP). cAMP is a mediator in intracellular signaling pathways^(10)^. The most common stimulation effect of caffeine is causing the increase of cAMP values in neurons and the discharge of neurotransmitters^(10)^. In some publications, it was suggested that caffeine might have a minimal effect on the menstrual cycle when taken as 300 mg/day or more, and could also stimulate ovulation^(7)^. A study performed on rats in puberty concerning the effects of caffeine on the ovary suggested that rather than indirectly, it increased estrogen release directly and inhibited the development of follicles^(25)^. It was stated that, with caffeine consumption, even though E_2_ levels increased, the effectiveness of estrogen in the target tissue decreased due to the direct effect of caffeine^(25)^. In another study, it was stated that high cAMP levels prevented germinal vesicle destruction and further oocyte maturation including polar body release^(10)^. Most PFs wait, dormant in the follicle pool, some develop, and proceed to the primary follicle phase^(20)^. In a study performed on postnatal rats, the number of PFs decreased in the caffeine-treated group and the size and number of secondary and AFs did not differ compared with the control group. This was attributed to the atresia of PFs due to the toxic effect of caffeine and to the replacement of developing secondary follicles with PFs^(26)^. In a subsequent study, the authors proposed the hypothesis that caffeine induced this damage by causing DNA damage in cells during cell division^(10)^. Caffeine has been reported to inhibit DNA repair and suppress meiosis in female mice^(23,27)^. In our study, no significant decrease was observed in the PF pool (p=0.439). We think that caffeine slows the growth of PFs. Although there is a decrease in AMH levels, we think that the insufficient growth of PFs is due to caffeine. This increased inhibition may cause cell damage in PFs in the long term.

Taurine is an important amino acid that has many functions in the body and is found in many organs^(8)^. Taurine is found in oocytes, granulosa, and theca cells, especially in the epithelial cells of the ovary and uterus. It was reported that cells achieve this through cysteine sulfinic acid decarboxylase^(28)^. However, there is mRNA-carrying taurine in the ovaries^(29)^. It was reported that in rats, taurine stimulated follicular development indirectly by the release of FSH, LH, and E_2 _through the hypothalamic-pituitary axis, or directly through E_2_ produced in granulosa cells by increasing androgen synthesis in the osmoregulator or theca cells^(30)^. In an *in vitro* study, it was reported that taurine could directly stimulate follicular development, as well as work as an osmotic regulator in embryos, mouse and human oocytes, and maintain the development of follicles and embryos^(31)^. In another *in vitro* study on rats, it was suggested that taurine directly stimulated the development of follicles through several ambiguous ways^(32)^. In an *in vitro* study on cattle, it was concluded that taurine was not useful in the embryonal development of bovine oocytes as a direct effect. In this study, we propose that the hormonal effect and growth affect the PSF follicle pool, which is the highest, the number of follicles due to stimulation in the PSF pool decreases (p=0.021) and accelerates the preovulatory oocyte passage with a decrease in AMH. We suggest that PFs, whose functions are inhibited, do not provide a reduced PSF pool despite the decrease in AMH, and taurine does not affect PFs because it has no intracellular function. In our study, the number of large AFs increased in subjects given ED. However, we suggest that taurine accelerated the development of oocytes that entered the growth cycle and increased the number of preovulatory oocytes due to the increased LF count (group I; 16±9.4, group II; 17.1±10.7), even though there was no statistical difference (p=0.283).

Some authors suggest that lipids are stored in adipose tissue as a result of increased lipogenesis due to excessive stimulation of insulin due to sugar components of EDs and increased carbohydrate metabolism^(33)^, and other authors suggested that the increased sugar level inhibited the satiety center and that there was weight gain due to the increase in energy intake^(34,35)^. However, no weight gain was observed in approximately 4-week studies on rats and rabbits^(36,37)^. In our study, significant weight gain was observed at the end of eight weeks in the ED group (p=0.002). However, a significant increase in food intake was also observed in the ED group (p=0.001). Therefore, like previous hypotheses^(33)^, we think that the sugar in the ED increases the level of insulin, causing an increase in catabolism and high lipid storage rates in adipose tissues, and that the increase in insulin level increases food consumption by causing a feeling of hunger. However, further studies are needed to explain this situation clearly.

### Study Limitations

This study is an animal experiment and there is no literature on the dose for ED use. Another difficulty of the study is that there are many components in EDs. The limitation of our study in the histologic examination is that immunohistochemical staining showing PF activity could not be performed. The effect of EDs on fertility could not be evaluated because the reproduction of the rats during the study period could not be controlled.

## Conclusion

The substances in EDs have a dominant effect on ovarian function in certain periods, for example, caffeine for inhibition, taurine as a stimulant and increased catabolism, and sugar for weight gain. However, we think that further studies are needed on how and in which ways they act separately and in combination for each substance in EDs.

## Figures and Tables

**Table 1:**
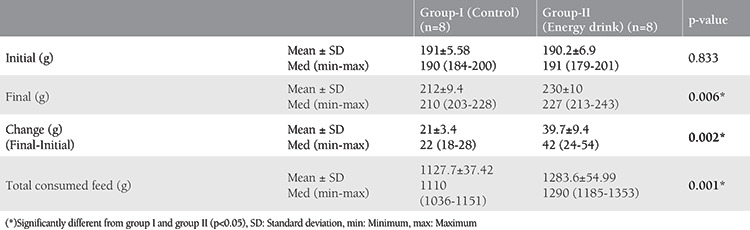
Means of weight changes between the groups

**Table 2:**
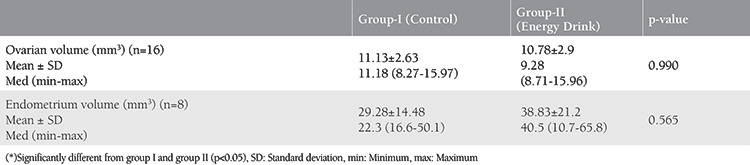
Comparison of ovarian and uterine volumes of two groups

**Table 3:**
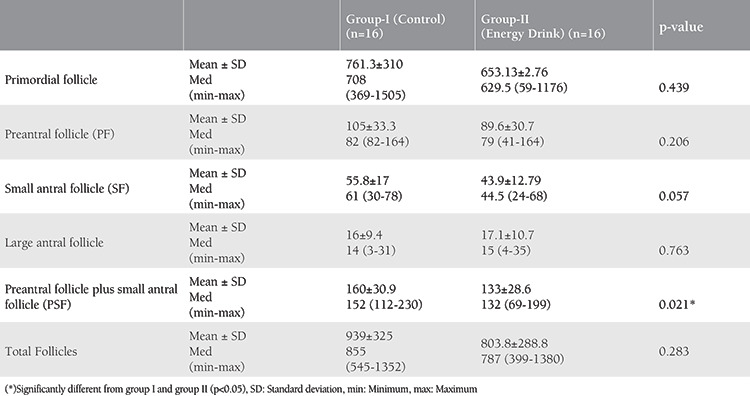
Classification and comparison of ovarian follicles in terms of dimensions

**Table 4:**
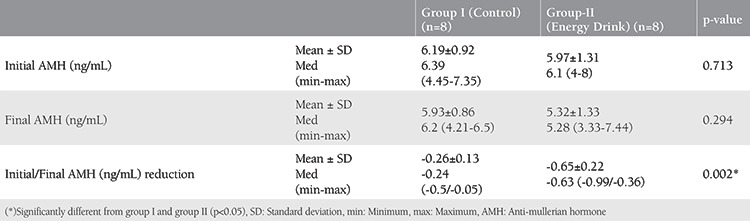
Comparison of AMH levels between the groups
